# Case report: Efficacy of lutetium-177 oxodotreotide for neuroendocrine tumor with central nervous system metastases

**DOI:** 10.3389/fnume.2023.1074948

**Published:** 2023-02-23

**Authors:** Nwanneka Okwundu, Christopher R. Weil, Heloisa P. Soares, Gabriel C. Fine, Donald M. Cannon

**Affiliations:** ^1^ Department of Radiation Oncology, University of Utah School of Medicine, Salt Lake City, UT, United States; ^2^ Department of Medical Oncology, University of Utah School of Medicine, Salt Lake City, UT, United States; ^3^ Department of Nuclear Medicine, University of Utah, Salt Lake City, UT, United States

**Keywords:** neuroendocrine tumor, lutetium (177Lu)-DOTA-octreotat (DOTATATE), central nervous system, metastases, peptide receptor radionuclide therapy (PRRT)

## Abstract

Neuroendocrine tumors (NETs) rarely metastasize to the brain. However, when they occur, NET brain metastases are associated with a poor prognosis. Due to their low incidence, NET brain metastases are poorly studied, with few data to guide a consensus for management. Prior reports have documented treatment with chemotherapy, resection, whole brain radiation therapy, and stereotactic radiosurgery, all with low rates of survival. We present a case of a patient with type 3 well-differentiated gastric NET with widespread metastatic disease, including central nervous system lesions in the pineal gland and left cerebellopontine angle (CPA), which were avid on ^68^Ga-dotatate positron emission tomography. The patient received four doses of 200 mCi (7.4 GBq) lutetium-177 oxodotreotide (^177^Lu-dotatate) administered every 8 weeks over the course of 6 months. The treatments provided local control of the pineal and CPA lesions for 23 months until the development of diffuse leptomeningeal progression that necessitated further therapies. ^177^Lu-dotatate may be a viable treatment for local control of NET brain metastases. More studies are needed to validate its efficacy in this clinical scenario.

## Introduction

Neuroendocrine neoplasms (NENs) are relatively uncommon heterogeneous neoplasms that are typically indolent (1–5). They make up about 2% of malignancies and are considered an orphan disease in the United States, with an incidence of 12,000 new diagnoses per year and a prevalence of <200,000 (6, 7). However, from the analysis of the Surveillance Epidemiology and End Results data from 1973 to 2012, the incidence has increased more than sixfold over the last four decades, possibly due to heightened awareness and improved imaging techniques. There was also a notable increase in prevalence from 0.006% in 1993 to 0.048% in 2012 (*p* < 0.001) (7).

The treatment of NENs depends on the grade, extent of metastasis, and aggressiveness. Based on the 2019 WHO classifications, neuroendocrine neoplasms can be divided into two categories: morphologically well-differentiated neoplasms are labeled neuroendocrine tumors (NETs), while poorly differentiated neoplasms that still express neuroendocrine markers are labeled neuroendocrine carcinomas (NECs) (8, 9). For low- to intermediate-grade NETs with localized disease, surgery is typically the first-line treatment, as surgery represents the only curative strategy. Subsequent adjuvant therapy is debatable (10, 11). Patients commonly present with unresectable locally advanced primary tumors or with metastatic disease, in which case, systemic therapy and palliative metastasectomy (if feasible) are employed to inhibit tumor growth and palliate symptoms (11–13).

Many NETs express somatostatin receptors (SSTRs), which have been explored as targets for peptide receptor radionuclide therapy (PRRT). There is increasing evidence for the utility of the radionuclide-linked somatostatin analog lutetium-177-dotate (^177^Lu-dotatate). ^177^Lu is a radionuclide with a half-life of 6.76 days that undergoes β-minus decay, emitting both beta particles with a maximum energy of 0.497 MeV and maximum tissue penetrance of 2–2.5 mm, and abundant 208 and 113 keV gamma photons, permitting its use as both a therapeutic and diagnostic radiopharmaceutical (14). ^177^Lu-dotatate is formed by coupling ^177^Lu with the high-affinity somatostatin analog octreotate using the tetraxetan (DOTA) chelator molecule (15). ^177^Lu-dotatate was approved by the FDA in 2018 for the treatment of SSTR-positive gastroenteropancreatic neuroendocrine tumors (GEP-NETs) as a last-line therapy in the setting of disease progression on fixed-dose long-acting octreotide (16–19). Although ^177^Lu-dotatate has shown benefits in the treatment of NETs with extracranial metastases, there is no currently established benefit in NET patients with central nervous system (CNS) metastases, with limited data on CNS penetration (20, 21). Historically, there has been concern about the limited entry of somatostatin analogs into CNS neoplasms behind an intact blood–brain barrier (BBB) (22). Prior reports using somatostatin analogs linked with DOTA and radionuclides in primary brain tumors utilized direct injections into the tumor or resection cavity, with none utilizing intravenous administration (23–27). We present a case of a patient with a gastric NET primary with CNS metastases treated with ^177^Lu-dotatate.

## Case description

A 68-year-old man presented with right upper quadrant abdominal pain and was noted on CT to have multiple ill-defined enhancing masses in his right hepatic lobe, concerning for metastatic disease. A fluorine-18 (^18^F)-fluorodeoxyglucose (FDG)-PET/CT scan showed multiple poorly defined masses within the liver [maximum standard uptake value (SUVmax) 3.1], a paraaortic lymph node (SUVmax 3.3), sclerotic lesions in the sacrum and spine (SUVmax 2.7), and a thickened gastric wall mass in the fundus and body (SUVmax 3.1). Fine-needle aspiration of a right central hepatic mass showed cells that were AE1/3, cytokeratin, synaptophysin, and chromogranin strongly positive, Ki-67 of 5%, most consistent with a type 3, grade 2, well-differentiated NET. Esophagogastroduodenoscopy (EGD) demonstrated a large, fungating mass arising from the body of the gastric wall, with a biopsy also consistent with the NET cells seen in the liver specimen. The final diagnosis was a stage IV, type 3, grade 2 metastatic NET of the gastric wall.

## Treatment course

**Figure 1:**
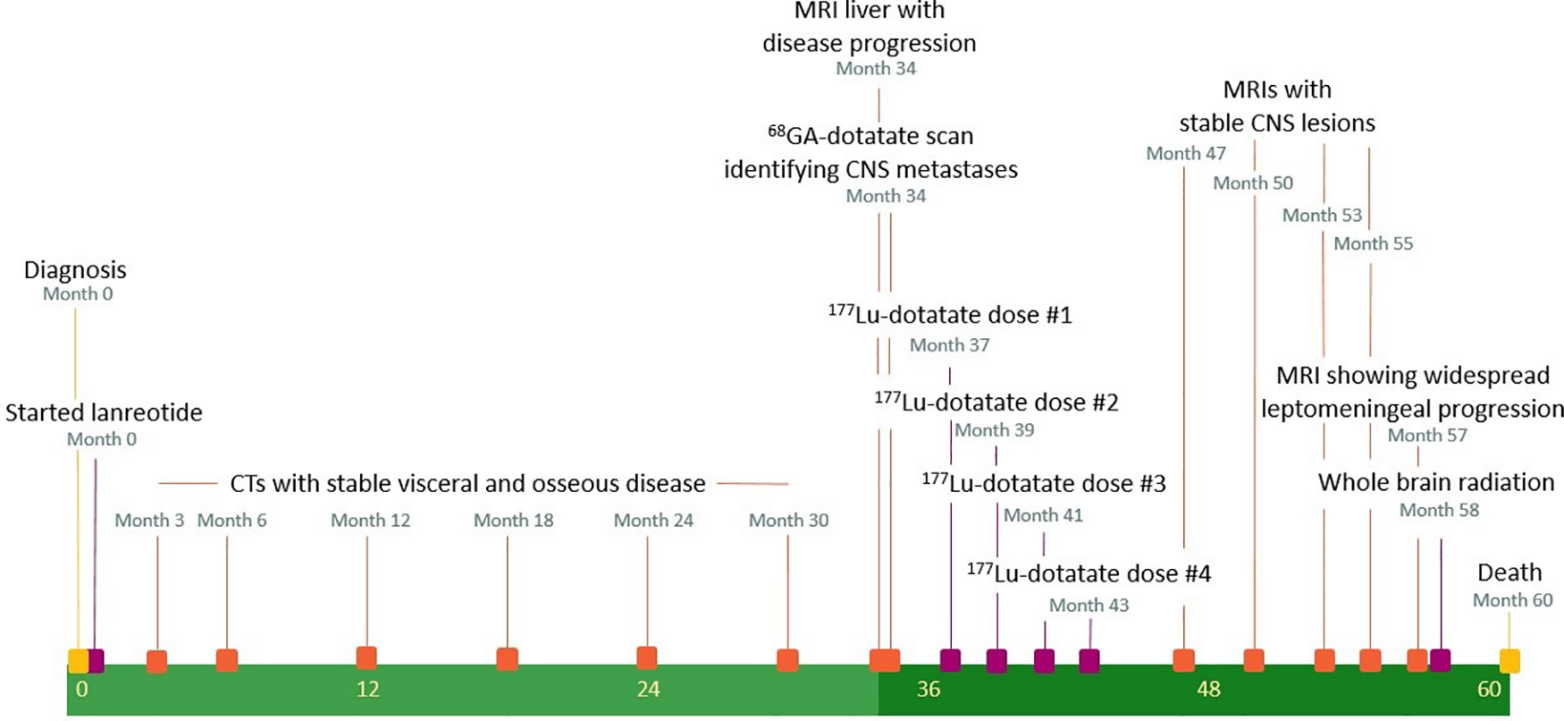
Timeline of patient's treatment course.

**Figure 2:**
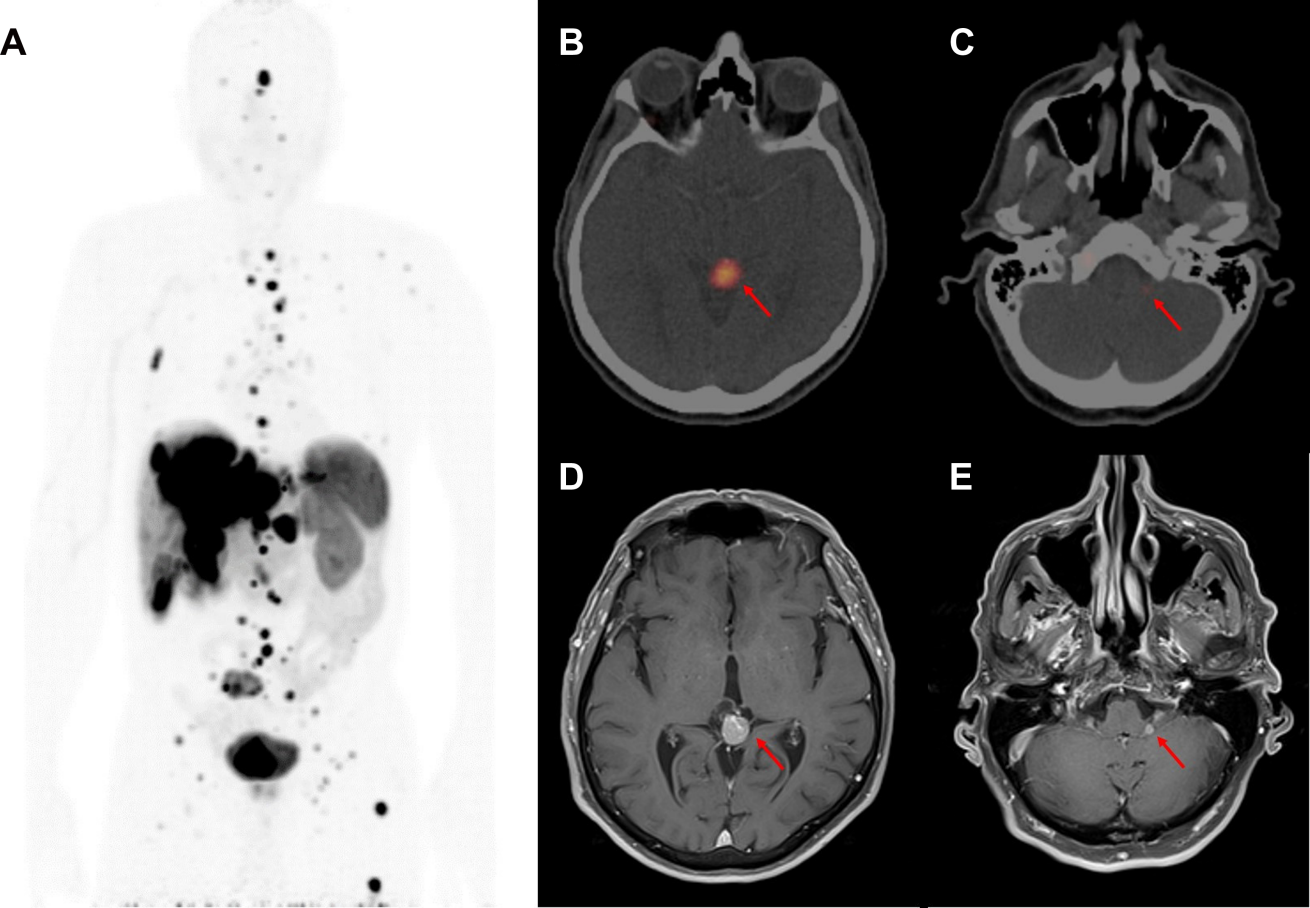
Initial diagnosis of central nervous system metastases at 34 months after diagnosis. (**A**) Inverted maximal intensity projection on coronal view from the ^68^Ga-dotatate PET/CT performed at 34 months after diagnosis showing the extent of metastatic disease expressing somatostatin receptors. (**B**) ^68^Ga-dotatate PET/CT showing an avid lesion in the pineal gland (SUVmax 36.4) measuring 1.3 cm × 1.1 cm. (**C**) ^68^Ga-dotatate PET/CT showing a small avid left cerebellopontine angle lesion (SUVmax 7.0). (**D**) MRI brain T1-post-gadolinium sequence performed at 35 months after diagnosis showing a 1.6 cm × 1.5 cm enhancing pineal mass. (**E**) MRI brain T1-post-gadolinium sequence showing a 0.5 cm × 0.4 cm left cerebellopontine angle enhancing lesion.

A timeline of medical interventions and patient response is shown in Figure 1. The patient was started on prolonged-release lanreotide at 120 mg every 4 weeks, which he took for 34 months, during which time he had stable but persistent disease according to imaging and repeated EGD biopsy. MRI of the liver at 34 months demonstrated an enlarging liver mass and a new retroperitoneal lymph node. At that time, he was referred to nuclear medicine for a gallium-68-dotatate (^68^Ga-dotatate) PET/CT scan and evaluation for PRRT with ^177^Lu-dotatate. The ^68^Ga-dotatate PET/CT showed uptake in the primary tumor in the stomach (SUVmax 22.4) widespread metastatic disease to the axial and appendicular skeleton (SUVmax 22.0), liver (SUVmax 42.2), and retroperitoneal and cervical lymph nodes (SUVmax 49.2), along with intracranial lesions at the left cerebellopontine angle (CPA) (SUVmax 7.0) and pineal gland (SUVmax 36.4). Maximal intensity projection from the ^68^Ga-dotatate PET/CT and fused axial images of the left CPA and pineal lesions are shown in Figure 2. The CPA and pineal uptake were concerning for leptomeningeal disease. Magnetic resonance imaging (MRI) of his brain redemonstrated lesions of 16 mm × 15 mm in the pineal region and 5 mm × 4 mm in the left CPA/foramen of Lushka, as demonstrated in Figure 2. An MRI scan of the cervical, thoracic, and lumbar spine was negative for spinal disease. A lumbar puncture was performed showing no evidence of malignant cells within the cerebrospinal fluid.

The patient was evaluated by radiation oncology for consideration of stereotactic radiosurgery (SRS) to the intracranial lesions. The patient was asymptomatic from both his intracranial and extracranial disease at that time, and there was concern for uncertainty regarding the combined radiation dose to the brainstem if the patient received both SRS and PRRT. As there was demonstrable uptake in the CNS lesions on ^68^Ga-dotatate PET/CT, an approach beginning with PRRT alone was favored in the hope of deferring SRS until a time of intracranial progression or development of focal neurological symptoms. Close CNS surveillance was planned as the extent of CNS penetration with ^177^Lu-dotatate was unclear. He received four total doses of ^177^Lu-dotatate over a 6-month interval, starting at 37 months from initial diagnosis (16, 17). The ^177^Lu-dotatate infusion was delivered over a 30-minute infusion prescribed at a target dose of 200 mCi (7.4 GBq), with measured delivered doses of 196.6, 197.2, 193.8, and 196.9 mCi, respectively. The ^177^Lu-dotatate infusions were delivered at 37, 39, 41, and 43 months from the initial diagnosis. He was treated with 8 mg of prophylactic ondansetron for nausea 30 minutes before each infusion, along with an intravenous amino acid infusion in the contralateral arm starting 30 minutes prior and continuing for an additional 4 hours after completion of the ^177^Lu-dotatate infusion for renal protection. He was also given 4 mg of prophylactic dexamethasone twice daily for 3 days due to concerns for treatment-related intracranial edema. All four infusions were tolerated without incident, and he did not develop any neurologic or hematologic toxicity after any of the four ^177^Lu-dotatate infusions.

**Figure 3:**
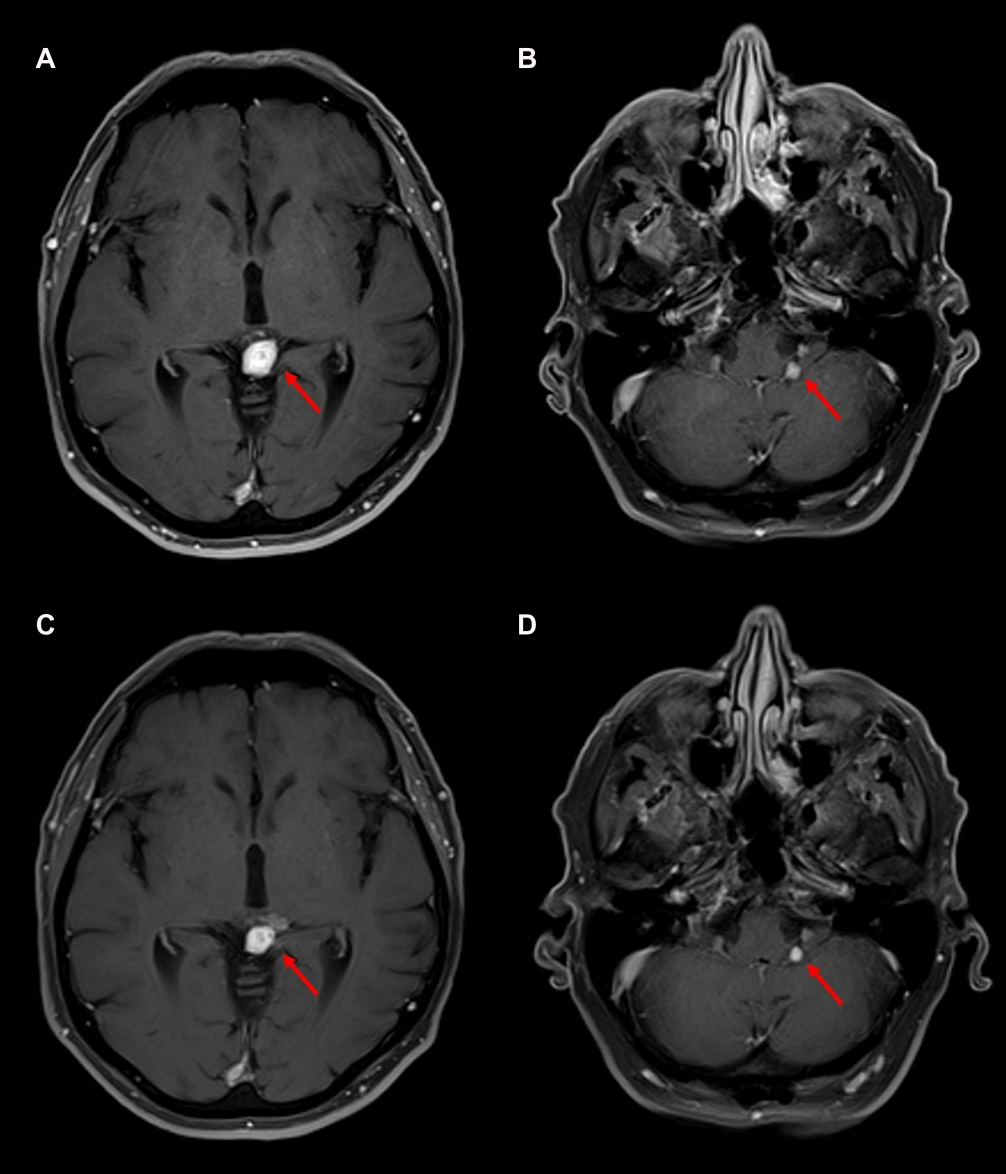
Follow-up MRI brain scans after four cycles of ^177^Lu-dotatate. T1-post-gadolinium sequences are shown. (**A**) MRI at 47 months after diagnosis, four months after completing ^177^Lu-dotatate, showing a 1.4 cm × 1.0 cm enhancing pineal mass, slightly decreased in size from diagnosis. (**B**) MRI 4 months after ^177^Lu-dotatate showing a 0.4 cm × 0.4 cm unchanged left cerebellopontine angle lesion. (**C**) MRI at 55 months after diagnosis, 12 months after completing ^177^Lu-dotatate, showing a 1.0 cm × 0.9 cm enhancing pineal mass, decreased in size but with some increased minimally enhancing cystic components adjacent but not extending from the pineal mass. (**D**) MRI 12 months after completing ^177^Lu-dotatate showing a 0.4 cm × 0.4 cm left cerebellopontine angle lesion, largely unchanged in size.

Both intracranial tumors had grown slightly on an MRI scan performed at 37 months from diagnosis, before his first PRRT treatment, with the pineal lesion measuring 19 mm × 17 mm and the left CPA lesion measuring 6 mm × 5 mm. An MRI of the brain before the third dose, performed 41 months after the diagnosis, showed a slightly increased lesion in the CPA at 7 mm × 6 mm and a decreased size and enhancement of the pineal lesion to 18 mm × 16 mm. After the fourth dose at 44 months, an MRI of the brain showed the pineal lesion had decreased further to 16 mm × 11 mm while the left CPA lesion remained essentially unchanged at 7 mm × 6 mm. Examples of axial MRI scans are shown in Figure 3, and lesion sizes over serial MRIs are shown in Table 1. CT scans of his chest, abdomen, and pelvis at 48 months after diagnosis, 5 months after the fourth dose of ^177^Lu-dotatate, showed complete resolution of the widespread hepatic metastases, decreased retroperitoneal lymphadenopathy, stable right adrenal, and stable, if somewhat increased, prominence of widespread osseous metastases. The patient did not have any neurologic or extracranial symptoms during this time.

**Table 1:** Largest axial dimensions of the pineal gland and left cerebellopontine angle lesions as measured by magnetic resonance imaging (MRI) using T1 post-gadolinium sequences, from the time of diagnosis until final MRI prior to death. The MRIs at 37, 41, and 44 months were performed within a few weeks of the patient's 1st, 3rd, and 4th dose of ^177^Lu-dotatate.

	Lesion size as read on MRI (mm)	
Months from diagnosis	Pineal	CPA
35	16 × 15	5 × 4
37	19 × 17	5 × 4
41	18 × 16	7 × 6
44	16 × 11	7 × 6
47	14 × 10	4 × 4
50	13 × 11	6 × 4
53	13 × 12	6 × 4
55	10 × 9	4 × 4
57	10 × 9	4 × 4

MRI, magnetic resonance imaging; mm, millimeter; CPA, cerebellopontine angle.

MRI of the brain continued to show no evidence of progression until 53 months from diagnosis, at which time he had new evidence of asymptomatic leptomeningeal disease in the left internal auditory canal (IAC); however, the CPA and pineal lesions remained unchanged. Repeat imaging at 55 months showed progression of the disease in the left IAC, facial nerve, and left dorsolateral brain stem. External beam radiation therapy (EBRT) to the base of the skull was offered, but he remained asymptomatic and so elected for treatment with capecitabine and temozolomide. CT scans of his chest, abdomen, and pelvis noted widespread progression of disease, with an increase in widespread sclerotic osseous lesions, numerous bilateral pulmonary nodules, and new and enlarging liver lesions and retroperitoneal adenopathy, though he remained clinically asymptomatic and continued with chemotherapy. However, at 57 months, he developed diplopia and gait instability, and an MRI scan demonstrated a left cerebellar peduncle hemorrhagic mass along with widespread leptomeningeal progression in the brain and spinal canal. He was seen by radiation oncology and elected to proceed with whole brain radiation therapy (WBRT) to 30 Gy in 10 fractions due to the extent of leptomeningeal disease. He ultimately succumbed to widespread progression of leptomeningeal and extracranial disease at 60 months from diagnosis, 23 months after the first ^177^Lu-dotatate infusion.

## Discussion

Metastatic NETs in different organ systems have been managed with variable success. Cranial metastases of NETs are both uncommon and portend a poor prognosis (28–31). Due to the rarity, the management of CNS metastases in this uncommon disease has not been well studied or reported, and there are no endorsed treatment guidelines. The limited data available reported 2- and 5-year overall survival rates of only 20% and 5%, respectively (32–34).

Reported cases have utilized a broad range of therapeutic interventions. These therapies include treatment with chemotherapy alone, resection, resection with adjuvant WBRT or SRS, or WBRT or SRS alone. The use of steroids is commonly reported for the symptomatic management of intracranial edema-related symptoms. The standard approach to treating functional NETs is with somatostatin analogs before other interventions in the setting of metastatic disease. PRRT plays an increasingly important role in the palliative management of treatment-resistant NETs due to its demonstrated improvements in response rates and progression-free survival in patients with metastatic or locally advanced disease, as demonstrated in the NETTER-1 trial (16, 17). However, the NETTER-1 trial did not specifically report on the number or outcomes of patients with CNS metastases, though they were allowed on trial, leaving the role of PRRT in these patients not well defined. In this particular patient, treatment with PRRT provided CNS control while allowing options for further CNS radiation therapy at the time of future progression. The treatment was well tolerated with minimal detriment to the patient’s quality of life.

This case report is novel as the patient showed sustained local control of CNS metastases in the pineal gland and left CPA, in addition to his extracranial disease, for a period of 18 months from the initiation of ^177^Lu-dotatate treatment. It could be argued that both of these lesions may not have been truly behind the BBB and, thus, activity by an intravenously administered systemic agent is not unexpected. The pineal gland is a circumventricular organ (CVO) with more permeable capillaries than vasculature in other parts of the CNS that are behind the BBB, suggesting possible access by systemically administered agents (35). A lesion in the foramen of Lushka could be leptomeningeal or, possibly, could arise from choroid plexus, which, while forming a tight barrier with the CSF, has highly fenestrated endothelial cells similar to CVOs (36, 37). From a practical standpoint, it may not always be necessary to distinguish whether disease is truly behind the BBB if clinical efficacy can be demonstrated. Even brain parenchymal metastases are characterized by the breakdown of the BBB, with responses seen by conventional cytotoxic chemotherapy agents, targeted small molecule agents such as EGFR tyrosine kinase inhibitors, antibody-drug conjugates, and immunotherapy (38–42). More studies are needed to further evaluate ^177^Lu-dotatate's CNS penetration and treatment efficacy in CNS metastases, both leptomeningeal and parenchymal.

This report also brings to mind the need for establishing workflows whereby accurate dosimetry can be obtained when radiopharmaceuticals, such as ^177^Lu-dotatate, are combined with EBRT treatments, such as SRS. For example, if upfront SRS had been pursued, understanding the cumulative dose delivered by PRRT to the brainstem near either the pineal or cerebellopontine lesions would be helpful in guiding the SRS prescription doses. Calculations of individualized dosimetry have been reported for ^177^Lu-dotatate but can be labor intensive and could benefit from standardization (43). However, such calculations would be beneficial for the integration of PRRT with EBRT in some cases, particularly in the CNS, to avoid possible toxicities associated with exceeding radiation tolerance to structures, such as the optic nerves or brainstem (44). Such integration may be important if there is a benefit to upfront EBRT, even in the setting of systemic therapies with CNS penetration, as suggested by one study in EGFR-mutated non-small cell lung cancer (45).

In conclusion, it has become apparent that PRRT with ^177^Lu-dotatate has already had a significant impact on the management of inoperable locally advanced or metastatic octreotide analog-resistant NETs. Though highlighting a rare clinical situation, this report indicates the potential for efficacy in NET patients with CNS metastases that show SSTR-targeted radiotracer uptake. Reporting on outcomes in a larger cohort could help establish the inclusion of patients with CNS metastases in future prospective studies of patients with NETs treated with PRRT.

## Data Availability

The original contributions presented in the study are included in the article/Supplementary Material, further inquiries can be directed to the corresponding author.
